# Brachial flow—mediated dilation and carotid intima—media thickness in glaucoma patients

**DOI:** 10.1186/s12886-022-02498-5

**Published:** 2022-06-23

**Authors:** Lovro Bojic, Veljko Rogosic, Domagoj Markovic, Lucija Vanjaka Rogosic, Duska Glavas

**Affiliations:** 1 Private Eye Clinic, Split, Croatia; 2 Department of Ophthalmology, University Hospital Split, Split, Croatia; 3 Clinic for Heart and Cardiovascular Diseases, University Hospital Split, Spinciceva 1, 21000 Split, Croatia; 4 School of Medicine, University of Split, Split, Croatia

**Keywords:** *Glaucoma*, *Flow-mediated dilation*, *Brachial artery*, *Carotid artery*, *Intima-media thickness*

## Abstract

**Background:**

The purpose of the study was to assess the ultrasound measurements of the brachial artery flow-mediated dilation (FMD) and carotid artery intima-media thickness (IMT) and their relationship in glaucoma patients.

**Methods:**

Thirty-seven patients with glaucoma and thirty-one healthy controls were included in the study. All glaucoma patients and controls underwent ultrasound measurement of FMD of the brachial artery and ultrasound measurement of IMT of the carotid artery.

**Results:**

The mean values of brachial FMD were significantly lower among the glaucoma compared with controls (16.4 ± 10.6% vs 20.3 ± 8.5%, *p* = 0.034). No significant difference was found in carotid IMT (1.2 ± 0.4 vs. 1.1 ± 0.4, *p* = 0.3), and brachial artery diameter at rest (4.7 ± 0.6 vs. 4.9 ± 0.3, *p* = 0.2) between the glaucoma patients and controls. The significant difference in brachial artery diameter in hyperemia between the glaucoma patients and controls (5.5 ± 0.6 vs. 5.9 ± 0.4 *p* = 0.002) was found. A negative correlation among brachial FMD and carotid IMT as well as among brachial FMD and brachial artery diameter at rest was found.

**Conclusions:**

Impaired brachial FMD indicates presence of systemic vascular endothelial dysfunction in glaucoma; glaucoma patients with lower values of the brachial FMD are at increased risk of having thickened carotid IMT.

## Background

Glaucoma is a multifactorial progressive optic neuropathy, characterized with acquired loss of retinal ganglion cells and their axons and atrophy of the optic nerve [1]. Glaucoma is one of the leading causes of irreversible blindness in the world [2]. The cause of glaucoma is still unclear today. Mechanical and vascular theory has been postulated as causative for glaucoma [3, 4]. Clinical treatment is focused on lowering intraocular pressure (IOP), although in some patients, glaucoma continues to progress despite well controlled IOP [5].

The vascular theory considers glaucomatous optic neuropathy (GON) as a consequence of insufficient ocular blood flow of the optic nerve head [6, 7]. Although ocular blood is often reduced because of elevated IOP, the existence of normal tension glaucoma suggest that other factors are also involved. Indeed, dysregulation of vascular resistance is now considered a key pathogenic factor. Moreover, dysregulation often manifest itself systemically as the primary vascular dysregulation syndrome. Vascular dysregulation leading to low perfusion because of vascular endothelial dysfunction play an important role in the pathogenesis of GON [8–10]. The primary vascular dysregulation can be the major cause of vascular endothelial dysfunction and formulated a concept of the GON [11–13]. In addition, some reports indicated that blood flow dysregulation are related to ocular blood flow disturbances in glaucoma [8, 9]. It has been shown that vascular endothelial function can be impaired in normal tension glaucoma (NTG) patients [10, 14]. Vascular endothelial dysfunction can be easily assessed by non-invasive ultrasound measurement of the flow-mediated vasodilation (FMD) of the brachial artery [15, 16]. FMD is a standard non-invasive repeatable and endothelium dependent technique that is used for an in vivo assessment of vascular endothelial functionality. FMD is useful for evaluation and follow-up of glaucoma patients. Recent studies have shown that glaucoma is associated with vascular endothelial dysfunction [17–20]. The ultrasound measurements of intima-media thickness (IMT) of the carotid artery may represent marker of cardiovascular events [21, 22]. The increased carotid IMT was found in glaucoma patients although in the Rotterdam study carotid IMT was not found to be associated with increased risk of glaucoma [23, 24]. Recent reports have shown that brachial FMD and carotid IMT are independent factors, but could be relatively modest predictors of coronary heart disease, although in the study of Simon there is no clear evidence that IMT measurements may improve coronary heart disease prediction [21, 22, 25].

So far, there are no studies that examined the relationship between the brachial FMD and carotid artery IMT in glaucoma. The aim of this study therefore was to assess the relationship between the brachial artery FMD and carotid artery IMT in glaucoma patients.

## Methods

The study enrolled 37 patients diagnosed with open angle glaucoma (18 men and 19 women) aged 64.7 ± 6.4 years, and 31 healthy controls (17 males and 14 females) aged 64.8 ± 8.1 years. The inclusion criteria for glaucoma patients were: IOP < 21 mmHg at the time of study, open anterior chamber angle on gonioscopy, glaucomatous optic disc cupping and visual field loss on Octopus perimetry. Glaucoma field defects were considered significant if there was a reduction in sensitivity of 5 dB or more. Visual field assessment was either conducted within 2 months from the recruitment or obtained from medical record, obtained no more than 6 months before the recruitment period. Patients who were diagnosed with any other type of glaucoma were excluded. Patients with underlying ocular conditions which may interfere with visual field interpretation were also excluded such as media opacities, cataract and vitro-retinal disease.

The normal healthy controls were recruited random selection from hospital staff or volunteers. The 31 healthy controls had normal physical and ocular examination and no history of cardiovascular disease. They were not using regular systemic medication. Visual field assessment in this group was not performed because ocular examination results were normal.

Exclusion criteria included patients with cardiovascular disease, systemic hypertension, dyslipidemia, diabetes mellitus, cerebrovascular disease, current smoking and patients currently taking antihypertensive drugs, nitrates, statins, aspirin and hormone replacement agents. No patients had migraine or Raynaud disease. The exclusion criteria were applied to both groups.

Patients with glaucoma received no systemic drugs, but one (31 patients) or a combination of up to two ocular hypotensive agents (6 patients) were discontinued 48 h before the ultrasound examination (in order to avoid the possible effects on test parameters).

### Assessment of FMD

Brachial artery FMD was assessed noninvasively by ultrasound examination as described previously [15–17]. Briefly, brachial artery ultrasonography (5.7 MHz linear transducer using a Vivid three Expert, ultrasound scanner, General Electric) was carried out in subjects after a 12 h fast and resting supine for at least 15 min in a quiet room.

The right brachial artery proximal to the antecubital fossa was imaged longitudinally using the linear–array transducer. The artery was longitudinally imaged 5 cm proximal to the antecubital crease, where the clearest image was obtained, and brachial artery diameter was measured. Brachial artery diameter was measured by B-mode ultrasound images at the end of diastole. The electrocardiography was recorded simultaneously to synchronize the image capture to the top of the R wave. Flow-mediated dilation was assessed by measuring the brachial artery diameter at baseline and during reactive hyperemia. Reactive hyperemia was induced by deflating a cuff previously inflated to 250 mmHg for 5 min in the forearm. The arterial diameter was measured at baseline and 45 to 60 s after cuff deflation from longitudinal images in which the lumen-intima interface was visualized on both, the interior and posterior walls.

FMD is expressed as the percent change in brachial artery diameter in response to hyperemia relative to baseline value.

FMD = VD (hyperemia) – VD (baseline) / VD (baseline) X 100%, where VD = vessel diameter.

### Assessment of IMT

The measurement of IMT of the carotid artery was described previously [21, 22]. Briefly, carotid ultrasound studies were performed with 5.7 MHz linear transducer using a Vivid three Expert, ultrasound scanner, General Electric. The image was focused on the posterior far wall of the left and right carotid artery. A minimum of 6 measurements of the common carotid far wall of the both sides were taken 10 mm proximal to the bifurcation to derive mean carotid IMT. The average of obtained values was taken as the IMT.

All measurements were carried out by the same investigator who was blinded to the characteristic of the subjects. Informed consent was obtained for each participant in the study, after the nature of the procedure has been fully explained.

### Statistical analysis

Statistical analysis was performed with Statistica for Windows (version 7.0. Stat Soft, USA). The Kolmogorov–Smirnov test was used to test normal distribution of all numerical variables. Continuous variables were compared between the groups by an independent samples Student´s t-test when normally distributed or by Man-Whitney U test when nonnormally distributed. Multiple regression analysis was used to analysis the relationship between single dependent variable (FMD) and several independent variables: IMT, brachial artery diameter and age. Correlations between variables were calculated by Pearson coefficient test. The chi-square test and Student ´s t-test to compare patient’s data such as gender and age were used. Findings with an error probability value of < 0.05 were considered to be statistically significant.

## Results

Using Studentʾs t-test and chi-square test for independence, we did not find a significant difference between patients with glaucoma and the healthy controls regard to age (t = -0.05; *p* = 0.95) and gender (chi-square = 1.09; *p* = 0.6). Baseline clinical characteristics of glaucoma patients and controls are shown in Table 1.

**Table 1:** Baseline clinical characteristics

	Glaucoma patients (*n* = 37)	Controls (*n* = 31)		*p* value
Age, y	64.7 ± 6.4	64.8 ± 8.1	0.95	
Gender, male/female	18/19	17/14	0.6	
IOP, mmHg	17.4 ± 1.0	15.2 ± 1.3	0.001	
C/D ratio	0.57 ± 0.14	0.27 ± 0.12	0.001	
Visual field, MD, dB	6.4 ± 0.87			
Type of medication				
Beta blockers	8			
Prostaglandin analogues	21			
Alpha-2 agonist	2			
Fixed combinations:				
Timolol maleate + dorzolamide	2			
Prostaglandin analogues + timolol maleate	4			

The results of the brachial artery ultrasound assessment of FMD, brachial artery diameter at rest, brachial artery diameter in response to hyperemia, and carotid IMT are presented in Table 2.

**Table 2:** Flow-mediated dilation (FMD) of the brachial artery, intima-media thickness (IMT) of the carotid artery and brachial artery diameter at rest and in response to hyperemia in patients with glaucoma and controls

	Glaucoma group	Control group	*p* value
No. of cases	37	31	
FMD (%)	16.4 ± 10.6	20.3 ± 8.5	0.034
IMT (mm)	1.2 ± 0.4	1.1 ± 0.4	0.3^a^
Brachial artery diameter at rest (mm)	4.7 ± 0.6	4.9 ± 0.3	0.2^a^
Brachial artery diameter -hyperemia (mm)	5.5 ± 0.6	5.9 ± 0.4	0.002

*P* < 0.05 statistically significant

Values are given as mean ± SD

^a^No significant

The mean values of FMD were significantly lower among the patients with glaucoma compared with controls (16.4 ± 10.6% vs 20.3 ± 8.5%, *p* = 0.034). No significant difference was found in carotid IMT (1.2 ± 0.4 vs. 1.1 ± 0.4 *p* = 0.3), and brachial artery diameter at rest (4.7 ± 0.6 vs. 4.9 ± 0.3, *p* = 0.2) between the glaucoma patients and controls. However, the significant difference was found in brachial artery diameter in hyperemia between the glaucoma patients and controls (5.5 ± 0.6 vs. 5.9 ± 0.4, *p* = 0.002).

The Pearson correlation analysis showed a negative correlation between brachial FMD and carotid IMT (*r* = -0.460; *p* < 0,001) as well as between FMD and brachial artery diameter at rest (*r* = -0.432 *p* < 0.001). FMD showed also a negative correlation with age (*r* = -0,408; *p* = 0.001). Multiple regression results showed a negative relationship between FMD as dependent variable and independent variables; IMT and brachial artery at rest (*F* = 13.3; *R* = 0,619; *p* < 0.001).

## Discussion

Glaucoma is multifactorial disease. The diagnosis, treatment and rate of progression is not solely associated with elevated intraocular pressure, although this pressure is only the most recognized treatable risk factor for developing progressive optic neuropathy [3, 4]. Recent reports in literature over the last years have shown on the possible role of vascular system and associated vascular mediators in glaucoma [3, 7]. Maintaining balance between nitric oxide (NO) and endothelin-1 (ET-1), as vascular regulators, is crucial. Vascular dysregulation appears to be as a consequence of imbalance between NO and ET-1. Considering the role of endothelium to maintain control of blood flow, it is quite possible that vascular dysregulation in glaucoma patients can be a consequence of vascular endothelial dysfunction [8, 9, 14, 18, 19].

A certain resemblance could be found also in retinal vascular function [26]. Local retinal endothelial structure and function can be assessed by static and dynamic retinal vessel analysis. New retinal imaging techniques confirm the pathogenic concept of vascular dysregulation in glaucoma [27, 28]. Changes in retinal vasculature were associated with optic disc damage and glaucoma [27, 28]. Such examinations reveal that retinal vessels are also key areas of interest in glaucomatous development. The correlation of retinal vessels function with large artery function is moderate at best. It needs for investigation of local retinal microvascular function in glaucoma patients in order to truly address the vascular theory behind GON. Vascular endothelial dysfunction can also be associated with the severity of glaucoma [29]. In our study, a sample size was too low to show any direct correlations. The ultrasound measurements of FMD of the brachial artery is the most common assessment tool of endothelial function and together with ultrasound measurements of IMT of the carotid artery may provide additional information on subclinical atherosclerosis and predict cardiovascular events [21, 22, 25]. In nowadays, FMD of the brachial artery is the gold standard for clinical examinations on conduit artery biology [30]. FMD is superior tool for assessment of cardiovascular risk [31]. In Shechter et al. study was shown that brachial artery FMD is independently predictor of cardiovascular events in relatively healthy subjects with no apparent heart disease [25].

Significantly lower FMD values of the brachial artery in glaucoma patients than in controls was found, confirming the results from previous studies dealing with vascular dysfunction in glaucoma patients, although measured FMD values in our study were generally higher than in other studies [10, 17, 19]. These differences can be explained by the accuracy of the ultrasound measurements. Ultrasound measurements of the brachial artery are technically challenging and measurements errors even in fraction of millimeters may influence on the accuracy of the examination [30, 32].

IMT as measure of atherosclerotic disease can be reliably determined by carotid ultrasound and could predict future cardiovascular and ischemic stroke incidence [33]. In early stage, glaucoma patients showed signs of subclinical vascular abnormalities; accent the need to consider circulation pathologies in the development of glaucoma [34]. Consistent with the evidence of impaired endothelial dysfunction in glaucoma, the impaired brachial FMD values, found in our study, showed a negative correlation with carotid IMT [17–20]. The classic cardiovascular risk factors such as diabetes mellitus, hypertension and dyslipidemia, known to be associated with increased carotid IMT, were excluded from the study. As the carotid IMT is recognized marker of carotid and coronary atherosclerosis, it is quite possible that our results could suggest that glaucoma patients may be at risk for carotid atherosclerosis, although no significant difference in carotid IMT between glaucoma patients and controls was found, as in Bossuyt et al. trial [35, 36]. Systemic vascular dysregulation and autonomic dysfunction can be important in glaucoma patients and could help us to extent the view on this disease [37].

In our case control study, we examined this relationship between brachial FMD and carotid IMT in glaucoma patients, which could not prove a causal relation between these variables. There are some limitations to our study. First, although the ultrasound measurements of brachial FMD and carotid IMT are noninvasive and highly reproducible techniques, these are also examiner dependent and, therefore, possible errors in measurements can influence on accuracy of the examination. Second, raises the question whether the brachial artery diameter at rest, found in this study, could influenced on our results-particularly FMD values, because it is well known that strongest predictor of FMD is baseline brachial diameter [38]. Also, the study was performed without prior sample size calculation. It is therefore explorative in nature and may have been underpowered for this less sensitive structural vascular biomarker such as carotid IMT. Finally, this study was conducted using a relatively small number of patients; our results should be confirmed by larger studies.

## Conclusions

Impaired brachial FMD indicates presence of vascular endothelial dysfunction in patients with glaucoma, which is negatively correlated with carotid IMT. Our data indicate that glaucoma patients with impaired FMD are at increased risk of having thickened carotid IMT, suggesting as a possible risk factor for cardiovascular disease.

## Data Availability

The datasets used and analysed during the current study are not available publicly because individual privacy could be compromised but are available from the author Lovro Bojic on reasonable request. The request can be made on email lovre.bojic@gmail.com.
